# Dynamic Fluid Flow Exacerbates the (Pro-)Inflammatory Effects of Aerosolised Engineered Nanomaterials In Vitro

**DOI:** 10.3390/nano12193431

**Published:** 2022-09-30

**Authors:** Kirsty Meldrum, Joana A. Moura, Shareen H. Doak, Martin J. D. Clift

**Affiliations:** In Vitro Toxicology Group, Swansea University Medical School, Swansea University, Wales SA2 8PP, UK

**Keywords:** in vitro, micro-fluidics, fluid flow, co-culture, lung, nanoparticles, aerosol exposure, quasi-ALI exposure

## Abstract

The majority of in vitro studies focusing upon particle–lung cell interactions use static models at an air–liquid interface (ALI). Advancing the physiological characteristics of such systems allows for closer resemblance of the human lung, in turn promoting 3R strategies. PATROLS (EU Horizon 2020 No. 760813) aimed to use a well-characterised in vitro model of the human alveolar epithelial barrier to determine how fluid-flow dynamics would impact the outputs of the model following particle exposure. Using the QuasiVivo^TM^ (Kirkstall Ltd., York, UK) system, fluid-flow conditions were applied to an A549 + dTHP-1 cell co-culture model cultured at the ALI. DQ_12_ and TiO_2_ (JRCNM01005a) were used as model particles to assess the in vitro systems’ sensitivity. Using a quasi- and aerosol (VitroCell Cloud12, VitroCell Systems, Waldkirch, Germany) exposure approach, cell cultures were exposed over 24 h at IVIVE concentrations of 1 and 10 (DQ_12_) and 1.4 and 10.4 (TiO_2_) µg/cm^2^, respectively. We compared static and fluid flow conditions after both these exposure methods. The co-culture was subsequently assessed for its viability, membrane integrity and (pro-)inflammatory response (IL-8 and IL-6 production). The results suggested that the addition of fluid flow to this alveolar co-culture model can influence the viability, membrane integrity and inflammatory responses dependent on the particle type and exposure.

## 1. Introduction

Engineered nanoparticles (ENPs) are particles that can be defined as having a diameter > 100 nm [1,2,3]. These particles are small enough to enter the cell without assistance and potentially inhibit normal homeostatic processes within the cells [1]. The size of ENPs relates to a higher surface (area) to volume ratio when compared to the same particles with a diameter > 100 nm [4]. Both surface area and reactivity have the potential to cause an (pro-)inflammatory response in vivo and in vitro [5,6,7,8]. Further, specific individual properties of ENPs (e.g., size, particle diameter and polydispersity) have the potential to influence their cellular interaction [9]. Therefore, it is important to consider different types of particles and elucidate how the cellular responses may change when exposed to different particles with different physical and chemical characteristics. 

A major route of exposure to humans of ENPs is inhalation, and therefore the lungs are vitally important in determining the various potential hazards of ENPs [10]. The alveolar region is of particular interest for ENPs as they are readily deposited within this region of the lung due to their aerodynamic size [11,12,13,14]. One of the most commonly used cell lines for this region in vitro is the A549 epithelial cell line [15]. Although it is known that this cell line does have its pitfalls [16], they have similar characteristics to the alveolar epithelial type II cells (ATII) [17,18]. ATII cells are also responsible for producing lung surfactants and are known to differentiate to type I epithelial cells in order to help facilitate repair to damage of the alveolar region [19], and therefore are a key epithelial cell in the homeostasis of the alveolar epithelium. As well as implementing the use of relevant epithelial cells to make an in vitro model, there also needs to be the addition of immune cells present within the region [17]. Macrophages are sentinel cells [20] and are an important part of the innate immune response within the lungs (i.e., they are the first cellular defence against inhaled xenobiotics) [21]. Commonly, these cells are co-cultured with epithelial cells to produce an alveolar model in vitro [22,23,24]. Models such as these have previously been used to assist investigations into the toxicokinetics of ENPs [25].

When using such in vitro systems, it is imperative to consider the physiological relevance of the model. It has been previously identified that the addition of differentiated THP-1 (dTHP-1) cells to the A549 culture has an enhancing effect [22,26,27], with increases in inflammatory mediators when compared to monoculture systems. Another consideration is how the cells are grown and acclimatised to this environment before being exposed to any potential test material. Most early in vitro based studies focusing upon lung cell cultures exposed to ENPs were performed under submerged conditions with the ENP added directly into the cell-culture medium [28,29,30]. However, the alveolar region is not a monolayer of cells under fluid, it is constructed of bio-fluid (e.g., surfactant) and cell layers exposed to air [31]. Lung surfactant has the potential to alter the effects of ENP exposed to the cells [30,31,32], therefore, it is important to implement cell types (e.g., A549) that are able to mimic this scenario when cultured at the air–liquid interface (ALI) in this environment [32,33,34,35,36,37]. It has also been identified that cellular properties (lipid composition, surface morphology and surface tension) change when cultured at the ALI [38]. Therefore, further emphasising the need to use in vitro lung cell studies at the ALI. 

While a culture is at the ALI there are various exposure methods that can be utilised for toxicity testing of ENPs. These include the use of the quasi-ALI exposure method [39] and aerosol exposure (either wet [40] or dry aerosol exposure [41]). Careful consideration is required upon selection of these methods, due to the amount of material necessary and the availability for cellular endpoint testing. 

A further component when developing a more physiologically relevant model [42,43,44] of the alveolar region for ENP toxicity testing is the addition of fluid flow on the basal side of the membrane to mimic vascular flow [45,46,47]. These components have been combined in very low throughput, high physiological relevance models, such as lung-on-a-chip devices that can combine multiple cell types under microfluids [48]. Thus, it is important to consider all these aspects to develop a model that considers the area of the lung of interest, the relevant exposure method and the addition of physiological aspects in order to produce a “next level” system that is capable of investigating the potential impact of inhaled ENPs.

Therefore, the aim of this study, was to develop further an established static in vitro model used to test ENP aerosol toxicology to incorporate fluid-flow dynamics. This was achieved by implementing the QuasiVivo^TM^ (Kirkstall Ltd., York, UK) system with a characterised A459_dTHP-1 coculture exposed (e.g., Vitrocell Cloud12, VitroCell Systems, Waldkirch, Germany) to different particle samples at the ALI. It is hypothesised that the addition of fluid flow would increase the sensitivity of the model compared to the static environment, but the exposure method (i.e., quasi-ALI vs. aerosol exposure) would have no impact on the response to ENPs.

## 2. Materials and Methods

All chemicals and reagents were purchased from Sigma Aldrich (UK) unless otherwise stated.

*Cell Cultures*

A549 (ATCC^®^ CCL-185^™^) cells were obtained from American Tissue Culture Collection (ATCC, Middlesex, UK) and were cultured at 37 °C in 5% CO_2_. A549 were cultivated in RPMI-1640 medium (Gibco, Paisley, UK) supplemented with 10% heat inactivated foetal bovine serum (FBS; Gibco, Paisley, UK), 2 mM L-Glutamine (Gibco, Paisley, UK), 100 U/mL penicillin and 100 µg/mL streptomycin (Gibco, Paisley, UK), cited as complete cell culture medium (CCM). Cells were passaged when ~80% confluent and used between passages 9–21 for all experimentation.

THP-1 (ATCC^®^ TIB-202^™^) cells were obtained from American Tissue Culture Collection (ATCC, Middlesex, UK) and were cultured at 37 °C in 5% CO_2_. THP-1 were cultivated in the above CCM. Cells were maintained within cultures of 1 × 10^6^ cells/mL and used between passages 5–12 for all experimentation.

A549 cells were seeded on the apical side of a Falcon cell culture insert (transparent PET membrane with 3 µm pores; Corning, Flintshire, UK, 12-well or 24-well size) at a density of 2.78 × 10^5^ cells/cm^2^ in CCM (500 and 100 µL, respectively) with CCM in the basal compartment (1.5 mL and 500 µL, respectively). Specifically, 12-well transwell inserts were implemented for static conditions while 24-well transwell inserts were implemented for the fluid flow conditions (as they are the only ones that will fit within the QuasiVivo^TM^ system). On the 4th day (i.e., 96 h) after cell seeding, the medium was changed and cells were switched to the air–liquid interface (ALI) with CCM in the basal compartment and apical compartment exposed to air (i.e., no medium on the apical layer) [49]. After switching to the ALI, epithelial cells were provided for 24 h to habituate to the new culture environment prior to ENP exposures [50,51,52]. 

In parallel, THP-1 cells were differentiated into a macrophage-like phenotype (dTHP-1) by incubating with 20 mM phorbol 12-myristate-13-acetate (PMA) for 48 h with a further 48 h of recovery in CCM. The medium was removed from the apical side of the epithelial culture and dTHP-1 cells were seeded onto the epithelial cells. The co-cultures were then incubated for 2 h (to allow for adherence of the macrophage cells to the epithelial cell layer) prior to the apical medium being removed (and the whole co-culture being switched to an ALI). The co-culture was then incubated at 37 °C for 24 h (included in the 48 h recovery phase of the dTHP-1 cells) before exposure.

*Fluid Flow System*

A QuasiVivo 600 system (Kirkstall Ltd., York, UK) was used, connected to a peristaltic pump (Parker pump model PF22X0103) with a flow rate of 0.32–0.4 mL/min. The system consists of three bioreactors and a media reservoir connected by tubes. The peristaltic pump circulates the media and was positioned after the media reservoir and before the bioreactors. Each bioreactor fits a 24-well transwell, where the co-culture is established. The three technical repeats are positioned in series, sharing the media. The total fluid circulating is 30 mL (note that this media is shared for the 3 technical repeats).

The three biological repeats (n = 3) are three independent experiments of the system described above.

*Characterisation*

The co-culture was grown until the switch to ALI before it was either placed in static conditions or fluid flow conditions. It was then characterised at ALI days one through to four to characterise any changes that the addition of fluidics to the model may induce. The same biochemical analyses were completed for characterisation as post ENP exposure. The monoculture was previously characterised as previously outlined Barosova, Meldrum [49].

*ENP*

TiO_2_ (JRCNM01005a) were supplied by the European Commission Joint Research Centre Nanomaterial Repository (https://ec.europa.eu/jrc/en/scientific-tool/jrc-nanomaterials-repository (accessed on 23 August 2021)). The specific physical and chemical characteristics have previously been reported by [53] (Table 1). 

DQ_12_ particles [54] were kindly donated by the Institute of Occupational Medicine (IOM), Edinburgh, and previously characterised [54,55] (Table 1). A previous study was also completed to determine if activated DQ_12_ particles [25] would be more biologically reactive, however they were not, so historical particles were implemented within this study. 

**Table 1:** Summary of primary characterisation data [53,54,56].

	*Z-Average* (nm)	*Polydispersity Index* (*PDI*)	*BET Surface* m^2^/g
*TiO*_2_	125.4	0.171	46.175
*DQ*_12_	720	0.52	10.1

All particles were dispersed and sonicated based on “The NANOGENOTOX dispersion protocol” (https://www.anses.fr/fr/system/files/nanogenotox_deliverable_5.pdf, accessed on 23 August 2021). Particles were dispersed by sonication (Branson Sonifier 250, Ø 13 mm, 400 W output power, 20 kHz) in sterile water. A stock suspension of ENPs was prepared at a concentration of 2.56 mg/mL, which was diluted in sterile water [25] to the desired concentration. All exposure concentrations, previously determined via IVIVE approaches (https://www.patrols-h2020.eu/publications/sops/index.php and https://www.patrols-h2020.eu/publications/sops/SOP-library-pdfs/3105_PATROLS-Guidance-Document-for-ENMs-lung-dosing-consideration.pdf?m=1636040473&, accessed on 23 August 2021), have been previously found to induce significant inflammatory effects in vivo (0–5.2 and 0–1 µg/cm^2^).

*Particle Exposures*

**Figure 1:**
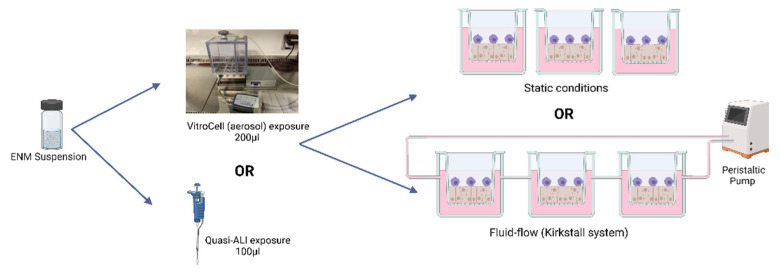
Exposure scenarios used. An exposure of an ENM is completed and analysed 24 h after exposure *via* either the quasi-ALI exposure or the VitroCell (aerosol) exposure method. Cultures are either in static conditions or under fluid-flow conditions using a peristaltic pump and the Kirkstall QuasiVivo^TM^ exposure system. All exposures equate to the same deposited exposure concentration regardless of the exposure scenario chosen. Cells are maintained at 37 °C and 5% CO_2_. Created with BioRender.com (accessed on 20 July 2022).

Cells were exposed either via a quasi-ALI exposure technique formally described in (39) for the static conditions, or through an aerosol exposure (VitroCell Cloud12, VitroCell Systems, Waldkirch, Germany), they were then placed in either static conditions or under fluid flow conditions (QuasiVivo^TM^ System (Kirkstall Ltd., York, UK)). Figure 1 outlines the various exposure approaches and conditions. For the quasi-ALI exposure method, this approach allows an accessible ALI exposure approach for when there are no aerosol exposure systems available [57]. This method entails exposing the cells apically to 100 µL of the particle suspension using a 6-well plate setup. In respect to aerosol exposures, co-cultures were exposed *via* the VitroCell Cloud12 (VitroCell Systems, Waldkirch, Germany) exposure system which entails exposing the cells apically to 200 µL of the particle suspension which is then nebulized and deposited. Real-time measurement of deposited particles is acquired *via* the use of a quartz microbalance (QCM) (40). For the higher concentrations, repeat exposures were required until the desired deposited concentration was reached. Vehicle controls (sterile water spiked with 1% NaCl) for both the low and high exposures (single and repeat controls) were completed as well as incubator negative and assay positive controls (specific to each biological assay kit and endpoint). 

Post exposure, static conditions were obtained by placing the transwells from the aerosol exposure chamber back into the companion plates and then placed into the incubator. Fluid flow conditions were established using the QuasiVivo^TM^ exposure system and pumping medium around (0.32–0.4 µL/min) the system with a reservoir volume of 30 mL. For each exposure implementing fluid flow conditions, three transwells were used due to the limitations in cell growth area (and therefore the number of cells for endpoint analysis).

Endpoint analyses were completed after 24 h of single exposures in both static and fluid flow conditions as well as after quasi-ALI and VitroCell exposures at 5% CO_2_ and 37 °C. With both systems, additional controls need to be considered to confirm that any effects identified within these studies are dependent on the particulate exposure and not the changes in humidity or temperature [58]. These controls included incubator controls (where the cells remained within the incubator instead of being placed into the VitroCell exposure chamber) and negative controls (including repeat exposures when the ENPs required multiple exposures to reach the required deposition concentration).

*Biochemical Analysis*

All samples from both exposure systems were processed for viability assessment (Trypan blue exclusion assay), whilst supernatants were collected and stored at −80 °C for future investigation of specific (pro-)inflammatory mediators.

For the comparison between fluid-flow and static, the differences in medium volume used were taken into consideration and corrected for as well as ensuring the cells on the different sizes of inserts were calculated in cells/cm^2^.

*Trypan Blue Exclusion Assay*

Cellular viability was determined using the trypan blue exclusion assay. Briefly, 10 µL of trypan blue dye (0.4%) was added to 10 µL of the cell suspension, before being counted with a haemocytometer and percentage viability calculated [49].

*Blue Dextran—Membrane Integrity Analysis*

The membrane integrity was analysed by measuring the translocation of Blue Dextran dye (Mw 200 kDa, Sigma Aldrich, Dorset, UK) from the apical to the basal compartment of the cultures after exposure to the particles. The translocation of Blue Dextran in an empty transwell (with no cells) was used as the negative control and results normalised against this. All medium was removed from the culture (leaving the cells on the membrane) before adding CCM to the basal side and 0.5% Blue Dextran (dissolved in PBS) added to the apical surface. The cultures were then incubated for 2 h at 37 °C before measuring the concentration of blue dextran in the basal compartment by measuring the absorbance values at 600 nm. This value was then shown as fold over the control value.

*(Pro-)Inflammatory Response*

The (pro-)inflammatory response of the cells following exposure to DQ_12_, and TiO_2_ was measured by quantifying the amount of the (pro-)inflammatory mediators released into the basal medium *via* Enzyme-Linked Immunosorbent Assay (ELISA). Lipopolysaccharide (LPS) (from *Escherichia coli*) was used as a positive (pro)-inflammatory control at 1 μg/mL on the basal side of the culture for 24 h. Cell culture supernatant was collected 24 h after exposure and analysed for cytokine levels of IL-8 (Cat no. DY208) and IL-6 (Cat no. DY206) using DuoSet kits from R&D systems (Bio-techne, Abingdon, UK) according to the manufacturer’s instructions. Samples were analysed in triplicate from three independent experiments (n = 3) and absorbance was assessed at 450 nm with background correction at 570 nm. Extrapolation of protein concentration was carried out from a standard curve of known concentrations (IL-8 (0–2000 pg/mL) and IL-6 (0–200 pg/mL)).

*Statistical Analysis*

All data are presented as the mean ± standard deviation (SD). All endpoints were assessed following three independent cell cultures (n = 3), except the static quasi-ALI exposures which were n = 5 after following the exposure protocol outlined previously [25]. Statistical analyses were performed using GraphPad Prism 8 (GraphPad Software Inc., San Diego, CA, USA) software. A two-way analysis of variance (ANOVA) with a subsequent Tukey’s multiple comparisons test was performed for each endpoint. Results were considered significant if *p* < 0.05.

## 3. Results

### 3.1. Model Characterisation

**Figure 2:**
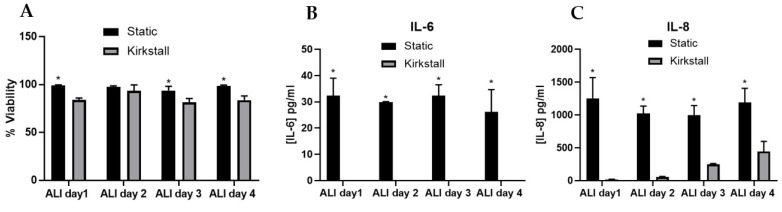
Characterisation of A549 + dTHP-1 co-cultures in both static (black bar) and under fluid flow conditions (grey bar). Cells were analysed at ALI days 1, 2, 3 and 4, focusing on cytotoxicity (**A**), IL-6 (**B**) and IL-8 (**C**) concentration. N = 3 with all assays performed in triplicate. The data is presented as the mean ± standard deviation. Significance is denoted as the following: compared to the fluid-flow equivalent *p* < 0.01 (*).

The addition of dynamic fluid flow to the model did significantly (*p* < 0.01) decrease the viability of the culture when compared to the static model (Figure 2A) on ALI days 1, 2, and 4, however, the viability of the model remained above 80% on every day analysed thereafter. For both IL-6 and IL-8 (Figure 2B,C), there was a significant decrease (*p* < 0.01) in the concentration in the fluid flow model when compared to the static culture. However, the IL-8 concentration does increase over the four-day period in the fluid flow model (though remained significantly decreased compared to the static model). The IL-6 concentration was not detectable in the A549_dTHP-1 co-culture under fluid flow conditions. The concentration of both IL-6 and IL-8 remained consistent (average within experimental variation) between ALI day 1 and day 4 analysed in the static model.

### 3.2. Quasi-ALI Exposure

Viability and Membrane Integrity

**Figure 3:**
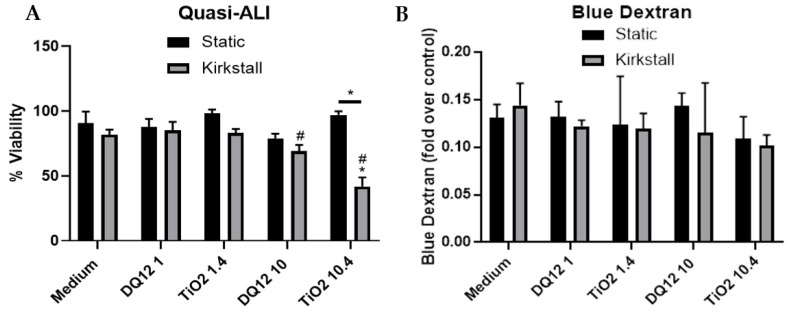
Viability and membrane integrity, 24 h post quasi-ALI exposure of both DQ_12_ and TiO_2_ on A549 + dTHP-1 co-cultures. Cells were exposed for 24 h at an ALI using the quasi-ALI method and incubated in either static or fluid flow conditions, before analysing cytotoxicity (**A**) and membrane integrity (blue dextran (**B**)). N = 3 for fluid flow and N = 5 for static with all assays performed in triplicate. The data is presented as the mean ± standard deviation. Significance is denoted as the following: compared to the medium control *p* < 0.01 (*); compared to the lower concentration *p* < 0.01 (#); line is comparing the two systems *p* < 0.01 (*).

The lower concentration of both DQ_12_ and TiO_2_ (1 and 1.4 µg/cm^2^) caused no changes in either the viability or membrane integrity of the A549 + dTHP-1 co-culture in either the static or the fluid flow model 24 h after a quasi-ALI exposure, when compared to the negative control (Figure 3A,B). However, there was a significant decrease in the viability (*p* < 0.05) after exposure to 10 µg/cm^2^ of DQ_12_ when compared to the lower DQ_12_ concentration and a significant decrease in the viability (*p* < 0.05) after exposure to 10.4 µg/cm^2^ TiO_2_ in the fluid flow model compared to the medium control, the lower TiO_2_ concentration, and the static equivalent (Figure 3A).

(Pro-)Inflammatory Response

**Figure 4:**
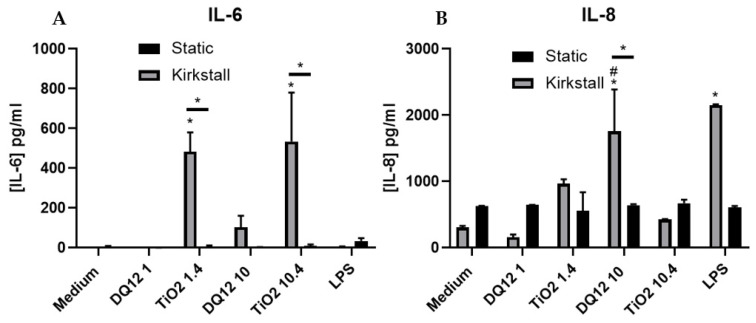
IL-6 and IL-8 basal concentration 24 h post quasi-ALI exposure of both DQ_12_ and TiO_2_ on A549 + dTHP-1 co-cultures. Cells were exposed for 24 h at an ALI using the quasi-ALI method and left to incubate in either static or fluid flow conditions, before analysing IL-6 (**A**) and IL-8 (**B**) concentrations in the basal compartment of the ALI culture after a single particle exposure (onto the apical side). N = 3 for fluid flow and N = 5 for static with all assays performed in triplicate. The data is presented as the mean ± standard deviation. Significance is denoted as the following: compared to the medium control *p* < 0.01 (*); compared to the lower concentration *p* < 0.01 (#); line is comparing the two systems *p* < 0.01 (*).

There was a significant increase (*p* < 0.05) in the concentration of IL-6 after exposure to both concentrations of TiO_2_ when compared to both the control and the static equivalent (Figure 4A). There was a slight increase (though not significant (*p* > 0.05)) in the IL-6 concentration after exposure to 10 µg/cm^2^ of DQ_12_ when compared to the negative control and the static equivalent. LPS exposure also did not cause significant increases in IL-6. There were no changes in the concentration of IL-8 after any of the exposures using the static model (Figure 4B). With the fluid flow model there was a significant increase (*p* < 0.01) in IL-8 after exposure to 10 µg/cm^2^ DQ_12_ when compared to the medium control, the lower DQ_12_ exposure and the static equivalent, as well as the exposure to LPS (*p* < 0.01). 

### 3.3. Aerosol Exposure

Viability and Membrane Integrity

**Figure 5:**
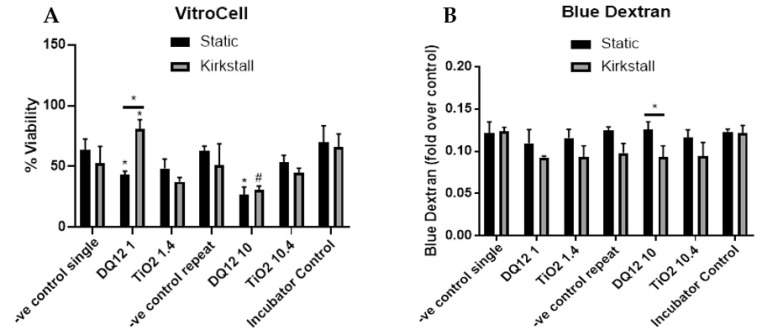
Viability and membrane integrity, 24 h post VitroCell exposure of both DQ_12_ and TiO_2_ on A549 + dTHP-1 co-cultures. Cells were exposed for 24 h at an ALI using the VitroCell method and incubated in either static or fluid-flow conditions, before analysing cytotoxicity (**A**) and membrane integrity (blue dextran) (**B**). N = 3 with all assays performed in triplicate. The data is presented as the mean ± standard deviation. Significance is denoted as the following: compared to the negative control *p* < 0.01 (*); compared to the lower concentration *p* < 0.01 (#); line is comparing the two systems *p* < 0.01 (*).

The introduction of the co-culture to the VitroCell exposure chamber did cause a decrease in the viability (~10% decrease) in both the single and repeat exposure controls (endpoints are measured at 24 h regardless) when compared to the incubator control, however, no significant changes (*p* > 0.05) in viability were noted (Figure 5A). There was a significant decrease (*p* < 0.05) in the viability of the co-culture after exposure to both DQ_12_ concentrations in the static model when compared to the appropriate control. Additionally, 10 µg/cm^2^ induced a significant decrease (*p* < 0.05) when compared to the fluid flow equivalent and the lowest DQ_12_ exposure. There was no change in the co-culture viability after TiO_2_ exposure in any of the physiological conditions (Figure 5A). There were no significant changes in the membrane integrity after any of the exposures when compared to the appropriate controls, however, there was a significant decrease in the membrane integrity induced by 10 µg/cm^2^ DQ_12_ exposure when comparing the fluid flow exposure to the static (Figure 5B).

(Pro-)inflammatory Response

**Figure 6:**
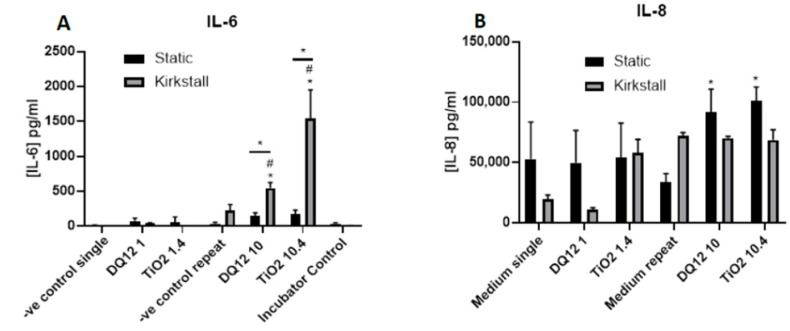
IL-6 and IL-8 basal concentration, 24 h post VitroCell exposure of both DQ_12_ and TiO_2_ on A549 + dTHP-1 co-cultures. Cells were exposed for 24 h at an ALI using the VitroCell method and left to incubate in either static or fluid-flow conditions, before analysing IL-6 (**A**) and IL-8 (**B**) concentrations in the basal compartment of the ALI culture after a single particle exposure (onto the apical side). N = 3 with all assays performed in triplicate. The data is presented as the mean ± standard deviation. Significance is denoted as the following: compared to the medium control *p* < 0.01 (*); compared to the lower concentration *p* < 0.01 (#); line is comparing the two systems *p* < 0.01 (*).

There was a significant increase (*p <* 0.05) in the IL-6 concentration after both the higher concentrations of DQ_12_ and TiO_2_ (10 and 10.4 µg/cm^2^, respectively) when compared to the appropriate negative control, the lower concentration, and the static equivalent (Figure 6A). There was no change in the IL-6 concentration after any of the exposures in the static model. There was an increase in IL-8 concentration after TiO_2_ exposure (1.4 µg/cm^2^) in the fluid flow model when compared to the negative control, but this was not significant (Figure 6B). There were significant increases (*p* < 0.05) in the IL-8 concentration after exposure to both DQ_12_ and TiO_2_ (10 and 10.4 µg/cm^2^, respectively) when compared to the negative control. 

## 4. Discussion

The aim of this study was to determine if the incorporation of dynamic fluid flow to an established static A549 + dTHP-1 co-culture model using both a quasi-ALI and wet aerosol exposure had the potential to increase the sensitivity of the model when compared to the static environment. After completing the characterisation of the co-culture it was indicated that the addition of fluid flow to the model system did not have detrimental effects (i.e., changes in viability and (pro-)inflammatory mediator release) to the static model (Table 2).

### 4.1. Addition of Dynamic Fluid Flow

The addition of fluid flow to the herein used in vitro lung cell model when applying the QuasiVivo^TM^ (Kirkstall Ltd., York, UK) was a straightforward way of implementing a model that was more physiologically relevant than traditional static in vitro models. Previously, using cells of the blood–brain barrier (i.e., astrocytes, pericytes and endothelial cells) with the fluid flow system, a slight decrease in cellular viability was identified in the fluid flow model when compared to the static model [59]. This was also identified within the current study, though with different cells from a different organ (i.e., the lung). This indicates that the implementation of fluid flow influences cellular viability within the initial 24 h period. From previous studies using the fluid flow model using an intestinal epithelium, it was identified that using the fluid flow system increases the barrier integrity and permeability, more closely mimicking the in vivo models [60]. This has also been identified with airway epithelial cells where the addition of fluid flow to the model led to an increase in the TEER value measured [61], which corresponds to the membrane integrity measured within this study (Figure 3B and Figure 5B) with blue dextran which was either slightly increased or remained the same when adding fluid flow. Previous work has identified that the TEER values previously measured have not matched what is found within the literature, however, these have increased when the A549-based models have been switched to an ALI [49]. Taken together, this suggests that the addition of fluid flow to the model has the potential to decrease the viability of multiple cell types when compared to the static controls, but the viability of the models remains consistently high, enabling the implementation of these systems for (ENP) toxicology testing. 

IL-8 is a neutrophil chemoattractant [62] with limited downstream effects, whereas IL-6 is a much more diverse cytokine and the continual production of IL-6 plays a role in chronic inflammation as well as tissue damage [63]. Both inflammatory mediators are produced by both epithelial and macrophage cells [64,65]. IL-6 links more so to chronic inflammation and lung epithelial damage. Due to the feedback loop of IL-6 [66], there is the potential that an initial release of this cytokine from the co-culture within the fluid flow system is then circulated around the system inducing the epithelial cells to further release IL-6, leading to the enhanced concentration found after both the quasi-ALI (Figure 3A) and the VitroCell (Figure 6A) exposures. In these exposures there is also a decrease in cellular viability which has been known to be linked to an increase in Il-6 production in specifically type II epithelial cells [67]. IL-6 has also been identified to protect A549 cultures from reactive oxygen species (ROS)-induced cell death [68], from our studies there is increased cytotoxicity after exposures paired with increased IL-6 production after the quasi-ALI exposures (Figure 3 and Figure 4A), this could potentially indicate that the cells are not dying due to ROS exposure, though future work is required to confirm this. This pattern is not identified in the VitroCell exposed cultures suggesting that this effect is relevant to the specific exposure method of ENPs to the in vitro cell culture. 

### 4.2. Quasi-ALI and Aerosol Exposures

The comparison of quasi-ALI and aerosol exposures has been previously completed within the literature, however, the addition of fluidics is something that is significantly lacking within the literature. Using the same TiO_2_ ENPs, at a very similar concentration (10.3 µg/cm^2^) there was no change in viability, however, at higher concentrations (such as 20.6 and 41.2 µg/cm^2^) a significant decrease in viability was induced after submerged exposures. However, this is a significant decrease at a much lower concentration (1.14 µg/cm^2^) when using an aerosol exposure [69], agreeing with what has been identified in the current study. Cellular uptake of TiO_2_ was found to be higher in submerged cultures of lung epithelial cells when compared to the aerosol exposure [70]. A549 cells grown at an air–liquid interface were previously exposed to silica nanoparticles (~50 nm) using both an “ALI deposition system (ALIDA)” (which implemented an electrostatic field and had higher deposition efficiency compared to the VitroCell) and a submerged exposure. It was determined that the submerged exposure elicited a stronger concentration of IL-8 when compared to the deposited particles [71]. This was not identified in our study; in fact the IL-8 concentration was ~100 times higher in the VitroCell exposures (Figure 6B) when compared to the quasi-ALI exposures (Figure 4B) in the static model and ~150 times higher in the fluid flow exposures (Figure 4B and Figure 6B) after exposure to both the highest concentrations of DQ_12_ and TiO_2_. This has also been identified using ZnO ENPs (at 1 µg/cm^2^) and an aerosol and “traditional” exposures using A549 cells [72]. The same was also true for IL-6 concentrations after exposure to the higher concentrations of DQ_12_ and TiO_2_ there was a ~5 and ~3 times increase, respectively, after VitroCell exposure compared to quasi-ALI exposures. However, this pattern was identified in the IL-6 concentration for the lower concentrations of DQ_12_ and TiO_2_, where more cytokine was released after quasi-ALI exposure compared to VitroCell exposure (Figure 4A and Figure 6A). It has been previously hypothesised [25] that IL-6 has the potential to be a better marker of a (pro-)inflammatory response after ENP exposures. This difference may also be related to the monoculture use previously [71], when compared to the use of a co-culture in this study. Other studies using the same TiO_2_ at similar concentrations (~1 µg/cm^2^) identified a significant increase in the concentration of IL-6, IL-8 and TNF-α post aerosolization when compared to the submerged control groups [73]. It has been previously identified that different exposure methods do have the capability to elicit different responses at different time points [74]. With changes to the exposure time and fractionated doses also contributing to these differences [25], endpoints must also be carefully planned due to potential degradation of the samples to be analysed [75].

It is important to note that the present study highlights the notion that the incorporation of additional physiological components may impact upon the biological responses (i.e., only the cytotoxicity and (pro-)inflammatory) tested, based upon the particle type and concentration as well as the exposure approach used. Further investigation is necessary, and ongoing, to confirm that the implementation of this physiological characteristic encourages the ability for such alternative systems to have the potential to predict the human health hazard of ENP inhalation exposure in a reproducible manner.

## 5. Conclusions

Taken together, it is evident that the addition of the physiological advancement (fluid flow) enhances the sensitivity of this co-culture (A549_dTHP-1) model when exposed to the specific particles at the herein tested concentrations, which is then further increased by implementing a more physiologically relevant exposure method (i.e., aerosolization of the particles, compared to quasi-ALI). However, with all things, the additions of these methods need to be balanced with the potential for the model to be implemented within other laboratories (both experience and monetary costs) and for the models to be further used as a part of the battery of models used for standard toxicological testing in the future. In conclusion, all future experimental work needs to consider the addition of physiological enhancements as well as exposure time and duration.

## Figures and Tables

**Table 2:** Summary of data. Trend is indicated by arrow and significance is indicated by appropriate symbol.

		Quasi-ALI						Aerosol					
		Static			Kirkstall			Static			Kirkstall		
		Viability	IL-8	IL-6	Viability	IL-8	IL-6	Viability	IL-8	IL-6	Viability	IL-8	IL-6
DQ_12_	1	↔	↔	↔	↔	↔	↔	↓*$	↔	↔	↑	↔	↔
	10	↔	↓*	↔	↔	↑#$	↑	↓$	↑$	↓*	↓#	↔	↑#$
TiO_2_	1.4	↔	↔	↓*	↓#	↔	↑$	↓	↔	↔	↓	↑	↔
	10.4	↑*	↔	↓*	↓#$	↔	↑$	↓	↑$	↓*	↓	↔	↑#$

* compared to Kirkstall system at the same concentration with the same particles; # compared to lower concentration; $ compared to negative control.

## Data Availability

The data presented in this study are available on request from the corresponding author. The data are not yet publicly available.
